# P03.06. Mind body interventions in medical education: a review of the literature

**DOI:** 10.1186/1472-6882-12-S1-P259

**Published:** 2012-06-12

**Authors:** A Natarajan, D Mehta

**Affiliations:** 1 Harvard Medical School, Boston, USA; 2 Benson-Henry Institute for Mind Body Medicine, Boston, USA

## Purpose

While many schools have made efforts to promote student wellness using various interventions, little is known about the impact. In 2000, Shapiro et al. conducted a literature review of stress reduction in medical education, and concluded the need for more rigorously defined studies. A decade later, there has been dramatic progress in understanding the deleterious consequences of stress on medical trainees. Given the sense of urgency to reform medical education, this study seeks to provide a descriptive review of studies examining mind body interventions in medical education.

## Methods

We searched Medline, Pubmed, PsychInfo, EMBASE, CINAHL, and ERIC for peer-reviewed primary studies of stress management interventions for medical trainees (medical students, interns, and residents) in the English language. The following subject heading terms and search strategy were used: (stress [psychological/prevention and control] or complementary therapies or spiritual therapies or spiritualism or religion or biofeedback or relaxation therapy or adaptation [psychological]) and (student, medical or education, medical).

## Results

Twenty-two studies met the eligibility criteria. There was great diversity in the types of interventions, combination of techniques, and frequency of meeting. Outcomes studied included stress, depression, anxiety, and overall mental health and quality of life. The majority of interventions show immediate value (either positive qualitative results from students) or positive improvements in outcome measures, but no studies examined if these results persisted over time.

## Conclusion

This review demonstrates the utility of a standardized mind body curriculum in medical education to buffer against the negative impacts of stress. Future research should focus on developing effective, evidence-based interventions alongside standardized instruments to examine medical student well-being. Future research may also seek to examine if multiple interventions provided at different points in training may serve as a buffer during these transition times.

